# Trigeminal neuralgia – a coherent cross-specialty management program

**DOI:** 10.1186/s10194-015-0550-4

**Published:** 2015-07-17

**Authors:** Tone Heinskou, Stine Maarbjerg, Per Rochat, Frauke Wolfram, Rigmor Højland Jensen, Lars Bendtsen

**Affiliations:** Danish Headache Center, Department of Neurology, Rigshospitalet Glostrup, Faculty of Health and Medical sciences, University of Copenhagen, Copenhagen, Denmark; Department of Diagnostics, Rigshospitalet Glostrup, Faculty of Health and Medical sciences, University of Copenhagen, Copenhagen, Denmark; Department of Neurosurgery, Rigshospitalet Blegdamsvej, Faculty of Health and Medical Sciences, University of Copenhagen, Copenhagen, Denmark

**Keywords:** Classical trigeminal neuralgia, Management program, Clinical care

## Abstract

**Background:**

Optimal management of patients with classical trigeminal neuralgia (TN) requires specific treatment programs and close collaboration between medical, radiological and surgical specialties. Organization of such treatment programs has never been described before. With this paper we aim to describe the implementation and feasibility of an accelerated cross-speciality management program, to describe the collaboration between the involved specialties and to report the patient flow during the first 2 years after implementation. Finally, we aim to stimulate discussions about optimal management of TN.

**Methods:**

Based on collaboration between neurologists, neuroradiologists and neurosurgeons a standardized program for TN was implemented in May 2012 at the Danish Headache Center (DHC). First out-patient visit and subsequent 3.0 Tesla MRI scan was booked in an accelerated manner. The MRI scan was performed according to a special TN protocol developed for this program. Patients initially referred to neurosurgery were re-directed to DHC for pre-surgical evaluation of diagnosis and optimization of medical treatment. Follow-up was 2 years with fixed visits where medical treatment and indication for neurosurgery was continuously evaluated. Scientific data was collected in a structured and prospective manner.

**Results:**

From May 2012 to April 2014, 130 patients entered the accelerated program. Waiting time for the first out-patient visit was 42 days. Ninety-four percent of the patients had a MRI performed according to the special protocol after a mean of 37 days. Within 2 years follow-up 35 % of the patients were referred to neurosurgery after a median time of 65 days. Five scientific papers describing demographics, clinical characteristics and neuroanatomical abnormalities were published.

**Conclusion:**

The described cross-speciality management program proved to be feasible and to have acceptable waiting times for referral and highly specialized work-up of TN patients in a public tertiary referral centre for headache and facial pain. Early high quality MRI ensured correct diagnosis and that the neurosurgeons had a standardized basis before decision-making on impending surgery. The program ensured that referral of the subgroup of patients in need for surgery was standardized, ensured continuous evaluation of the need for adjustments in pharmacological management and formed the basis for scientific research.

## Background

Classical trigeminal neuralgia (TN) is characterized by brief, very severe, shock-like pain paroxysms usually unilaterally in the 2nd and/or 3rd trigeminal branch [1, 2]. The diagnosis and management of TN traditionally lies in the hands of neurologists, anaesthesiologists, dentists, neurosurgeons and neuroradiologists. A close collaboration between these specialists would be ideal but is often complex and difficult to implement. Due to the relatively low lifetime prevalence of the disease of 0.3 % [3], the clinical experience even for pain specialists, is limited. Management of TN should be based on scientific evidence and clinical experience and be handled by experts as TN can be difficult to diagnose and treatment can cause troublesome side effects and complications [4–8]. Therefore, centralization leading to a high number of referrals is important because it allows sufficient experience for the treating clinicians [9]. Furthermore, a collaborative referral strategy between the involved specialties is important to ensure standardized and efficient management of the disease. To our knowledge, there are no previous papers describing the organization of TN patient care and how to implement a cross-speciality management program for TN.

The aims of TN management are: (a) to ensure that diagnosis is correct; (b) to exclude secondary causes by appropriate history, neuroimaging and clinical and laboratory examinations; (c) to optimize medical treatment and educate the patient in how to titrate medication up and down according to the level of pain and side-effects; (d) to decide whether, when and what type of neurosurgical intervention that should be performed and finally; (e) to prospectively collect scientific data about TN. To meet these requirements, a coherent accelerated management program for TN was implemented in May 2012 in a collaborative effort between three departments.

The aims of this paper are to describe the implementation and practical organization of this cross-speciality management program with particular emphasis on the collaboration between the specialties. Furthermore, we aim to report the flow of patients during the first 2 years after implementation of this practice. It is the overall aim to create scientific awareness of TN management and to inspire discussions about how TN ideally should be handled in clinical practice.

## Methods

The Danish Headache Center (DHC) is a public tertiary medical referral centre for headache and facial pain. It was established in 2001 and has since then received and treated headache and TN patients from all over Denmark.

With increasing clinical experience and collaboration with the Danish patient organization in TN “Trigeminus Foreningen” we recognized that the work-up, diagnosis, medical treatment and referral to and follow up after neurosurgery were highly variable and inconsistent both at our centre and nationally. This led to meetings with consultants from the Departments of Neuroradiology and Neurosurgery, to discuss how work-up, treatment and research could be optimized.

### Structure of the accelerated treatment program

**Fig. 1:**
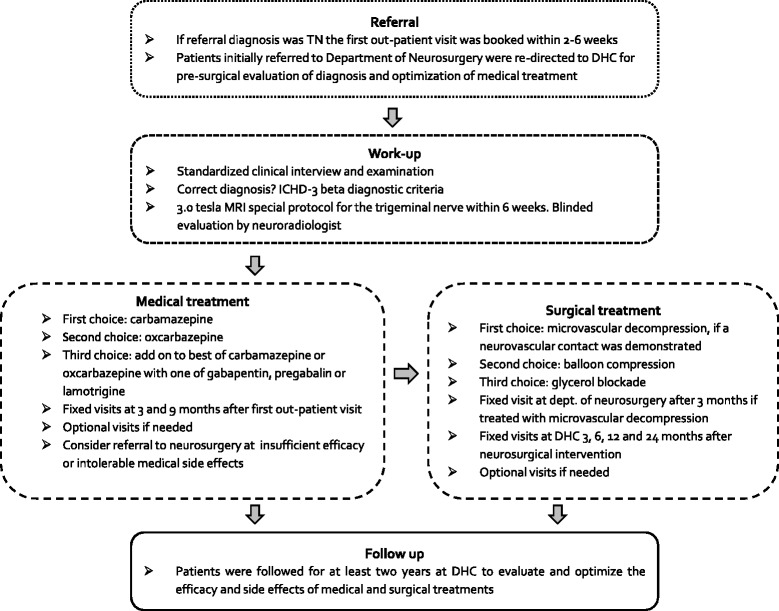
Algorithm of the accelerated work-up and treatment program. The three specialties involved in the work-up, diagnosis and treatment collaborate closely to secure a seamless patient path. TN: classical trigeminal neuralgia, DHC: Danish Headache Center, ICHD-3 beta: beta-version of the 3rd International Classification of Headache Disorders

In May 2012 we implemented an accelerated work-up and treatment program with a seamless patient path based on a formal collaboration between the neurologically staffed DHC (TH, SM, RHJ and LB), the Department of Radiology (FW) and the Department of Neurosurgery (PR). Patients were referred to DHC directly from general practitioners, private neurologists or hospital departments. Patients referred directly to the Department of Neurosurgery were re-directed to DHC for MRI and pre-surgical evaluation of diagnosis and medical treatment. The accelerated work-up and treatment program is outlined in Fig. 1.

Due to lack of resources the general waiting time for headache patients at DHC is up to 2 years. We therefore find it necessary to prioritize patients with the most severe or potential serious disorders such as TN, cluster headache and idiopathic intracranial hypertension. The aim was to see TN patients within 2–6 weeks after referral. As to patients re-directed from the Department of Neurosurgery the aim was to book their first out-patient visit within 2–4 weeks. At the first out-patient visit the previous diagnosis and treatment were re-evaluated. The diagnosis was initially based on the 2nd edition of the International Classification of Headache Disorders (ICHD-2) [10], but from June 2013 the diagnosis was based on the beta-version of ICHD-3 [1]. Categorical and quantitative variables were systematically collected in a semi-structured interview, designed for this TN management program. The interviewing clinician paid special attention to onset, periodicity, localization, character, intensity and frequency of pain and accompanying autonomic symptoms, to ensure correct diagnose. The patients were systematically questioned about previous treatments related to TN including; efficacy, duration, dosage and side effects of medical treatment and previous neurosurgical intervention. Scientific data was collected in a structured and prospective manner. It was confirmed by the Danish National Committee on Health Research Ethics ID number H-1-2012-093 that the project did not need ethical approval or patient’s informed consent.

The clinical examination included full routine clinical and neurological examination with special emphasis on trigeminal sensory function. A cotton swap and pin was used to examine touch and pinprick respectively, in all three branches of the trigeminal nerve. Examination of the intraoral sensory function of the mucosa of the cheeks and on either side of the tongue was tested with a cotton swap. Evaluation of the corneal reflex was also done systematically with a cotton swap. The majority of patients had already been thoroughly examined by a dentist to exclude odontogenic causes of pain. If not, patients were referred to their own dentist if this was considered relevant.

At the first out-patient visit, thorough oral and written information about the disease, its causes and potential medical and neurosurgical treatments were given. The written information was developed in collaboration between the involved neurologists and neurosurgeons. After the first visit patients were referred to a 3.0 Tesla MRI scan performed according to a special protocol [11, 12], designed to visualize the trigeminal nerve and its relations, and to identify symptomatic trigeminal neuralgia. The neuroradiologist described the MRI blinded to pain side. The aim was to perform the MRI within 4 weeks after the first out-patient visit.

### Medical treatment and the cross-speciality team

Medical treatment was adjusted at fixed out-patient visits at 3 and 9 months after the first visit and thereafter according to need. Phone consultations with trained headache nurses at DHC could also be booked and the nurses could book additional consultations with the doctors, if needed. Patients were followed for at least 2 years in order to optimize medical treatment and the need for referral to neurosurgical treatment was continuously evaluated. At each follow-up visit at DHC the treating neurologist performed a semi-structured interview, recording whether the patient was in remission, type and dose of medical treatment and its effects and side effects.

Patients were treated according to the international guidelines developed by the American Academy of Neurology and the European Federation of Neurological Societies [13]. First choice medical treatment was carbamazepine titrated to a dose sufficient to relieve pain or the maximum tolerated dose. Second choice was oxcarbazepine titrated in the same manner. If there was a response to carbamazepine or oxcarbazepine but unacceptable side effects at high doses, we used add-on treatments where first choice was gabapentin, then pregabalin and lamotrigine in sufficient and tolerable dosages. Only one combination treatment was tried and if the side effects to even low doses of carbamazepine or oxcarbazepine were unacceptable, we used gabapentin, pregabalin or lamotrigine, as monotherapy or in combination.

After pain freedom or insignificant pain for at least 1 month we encouraged patients to taper medication by one or half a tablet every seven to 14 days. In case of severe pain exacerbation that could not be controlled with oral treatments patients were admitted to in-patient treatment to optimize medical treatment. In severe cases patients were treated with intravenous loading of fosphenytoin.

We defined failed medical treatment as lack of efficacy or intolerable side effects to carbamazepine, oxcarbazepine and to a combination of the best of these drugs and gabapentin, pregabalin or lamotrigine. In such case neurosurgical treatment was suggested to the patient. Oral and written information about the efficacy and potential complications for each type of surgical intervention was given by the treating neurologist. Preferably, a close relative or a friend should accompany the patient during oral information. We focused the consultation on the patient’s current quality of life, pain intensity and on the medical side effects with respect to physical, social and psychological functions. This was weighed against the expected chance of a successful outcome of surgery and the potential surgical complications. Indication for surgery and its efficacy and potential complications were also discussed with the patient by the neurosurgeon, who was responsible for the final decision about surgery. Microvascular decompression was first choice surgical treatment when a neurovascular contact was demonstrated on the MRI and there were no contraindications to open neurosurgery. Second choice was percutaneous balloon compression. If neither microvascular decompression nor balloon compression did have effect percutaneous glycerol blockade was offered as third choice. Patients that were treated with microvascular decompression were routinely seen in the outpatient clinic at the Department of Neurosurgery 3 months after surgery. All neurosurgical treated patients were routinely seen in the outpatient clinic at DHC at standardized visits 3, 6, 12 and 24 months postoperatively to evaluate efficacy, complications, patient satisfaction and the need for medical treatment after surgery.

### Evaluation of the patient flow

To evaluate the feasibility of the implemented management program and the patient flow the first 2 years we describe the referral pattern of TN patients from May 2012 to April 2014. We evaluated the waiting time of referral and work-up and whether and when the patient was referred to neurosurgery within this period. Patients who had been treated at DHC before May 2012 were excluded as were patients with communication barriers and patients who for some reason did not enter the accelerated program.

### Statistical analyses

Continuous data are summarized by descriptive statistics. Categorical variables are presented with frequency distributions (N, %) and 95 % confidence intervals. Chi square and Fisher’s exact tests were used as appropriate to assess associations of categorical variables. P-values are reported as two-tailed with a significance level of 5 %. SAS 9.3 (SAS Institute, Inc., Cary, NC, USA) were used for all analyses.

## Results

**Fig. 2:**
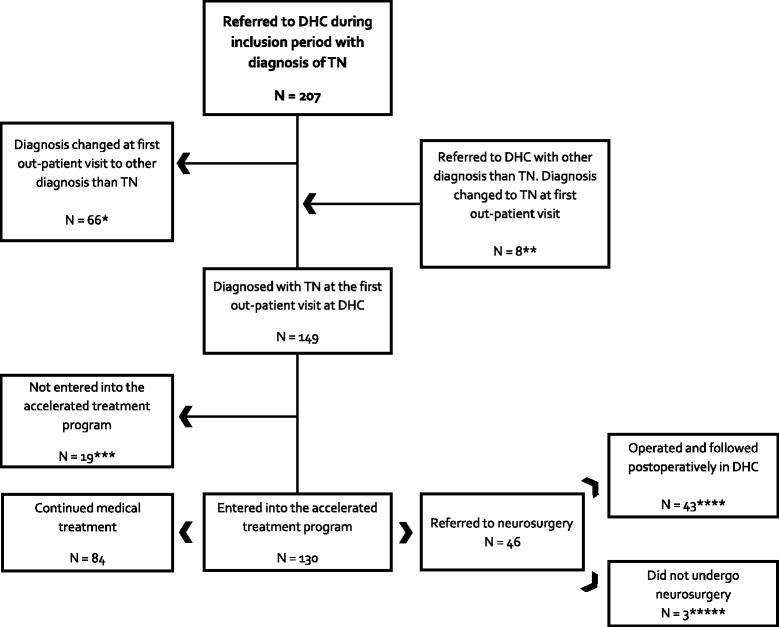
Flowchart of included patients. Inclusion period lasted from May 2012 to April 2014. TN: Classical trigeminal neuralgia, DHC: Danish Headache Center. *Diagnosis changed to: persistent idiopathic facial pain (PIFP) (*N* = 33), symptomatic trigeminal neuralgia (*N* = 12), cluster headache (*N* = 6), headache not elsewhere classified (*N* = 5), tension type headache (*N* = 3), migraine (*N* = 3), medication-overuse headache (*N* = 2), occipital neuralgia (*N* = 1), painful trigeminal neuropathy attributed to other disorder (*N* = 1). **Diagnosis changed from: PIFP (*N* = 4), tension type headache (*N* = 2), cluster headache (*N* = 1), headache not elsewhere classified (*N* = 1). ***Reasons for missing inclusion: pain free and did not want further controls (*N* = 7), unknown (*N* = 4), patient preferred treatment closer to home (*N* = 3), tumor not related to TN (*N* = 3), communication barrier ((Alzheimer’s) *N* = 1), death not related to TN (*N* = 1). **** Type of surgery: microvascular decompression (MVD) (*N* = 29), balloon compression (*N* = 10), both balloon compression and MVD within 12 months (N = 2), glycerol injection (*N* = 1), failed balloon compression due to bradycardia (*N* = 1). *****Did not undergo surgery due to: the neurosurgeon decided not to operate (*N* = 2), surgery was cancelled as the patient was pain free (*N* = 1)

Two hundred and seven patients with suspected TN were referred to DHC from May 2012 to April 2014 (Fig. 2). Sixty-six (31 %) patients were referred from general practitioners, 41 (19 %) from private neurologists and 68 (32 %) from other hospital departments. Forty (19 %) patients were re-directed from the Department of Neurosurgery. In 66 (31 %) patients the referral diagnosis of TN was not correct. Referral diagnosis was most frequently changed to persistent idiopathic facial pain (33 (50 %)), symptomatic trigeminal neuralgia (12 (18 %)) and cluster headache (6 (9 %)) (Fig. 2). Eight patients were referred to DHC with other diagnosis than TN and were diagnosed with TN at the first out-patient visit. Thus, 149 patients were diagnosed with TN in the inclusion period. Hereof 130 patients entered the accelerated treatment program. Nineteen (13 %) patients were not included in the accelerated program. Seven patients were pain free at their first visit and did not want further controls, four patients did not enter the program for unknown reasons, three patients preferred treatment closer to home, three had tumors not related to TN, one patient had Alzheimer’s and one died of cause unrelated to TN, before further follow-up.

Significantly more women (86 (66 %)) than men (44 (34 %)) entered the accelerated treatment program, p < 0.001. Mean age of disease onset was 55 (95 % CI 52.6 - 57.4) years and the mean age at referral was 62 (95 % CI 59.4–64.6) years. Mean duration of the disease was 7 (95 % CI 5.3–8.2) years. The median waiting time for the first out-patient visit was 42 days, range 6 to 154 days. For subsequent 3.0 Tesla MRI the median waiting time was 37 days, range 0 to 168 days. Data from 21 patients were not included in the analysis as 13 patients had their protocol MRI done before their first out-patient visit and 8 patients did not have MRI performed according to the special protocol.

### Referral to neurosurgery

Of the 130 TN patients who entered the accelerated treatment program 46 (35 %) were referred to neurosurgery after a median time of 65 days (range 0–588 days). Twenty-five of the 130 TN patients, who entered the program, were initially re-directed to DHC from the department of neurosurgery. Twelve (48 %) of these re-directed patients were referred back to neurosurgery after a median of 20 days (range 0–92 days) after their first out-patient visit. A total of 46 patients were referred to evaluation for surgery, 43 underwent surgery, hereof 29 (67 %) had microvascular decompression, 10 (24 %) balloon compression, two had both microvascular decompression and balloon compression, one glycerol blockade, while one failed balloon compression due to bradycardia. The three patients that were not operated continued medical treatment and follow-up at DHC.

### Medical treatment and follow-up

Eighty-four patients were not referred to neurosurgery but continued medical treatment at DHC. Hereof, 56 (67 %) patients were taking medications at their third medical follow up. Of the 28 patients who were not taking medicine at their third medical follow up, 19 (68 %) were in remission. The remaining 6 patients had tolerable pain and preferred to be without medicine. Data was missing in three patients. The median time of the third medical follow up was 308 days after the first outpatient visit. Thirty-five (63 %) of the patients who were taking medicine at their third medical follow up were receiving monotherapy with either carbamazepine or oxcarbazepine. Twelve (21 %) were taking gabapentin, pregabalin or a tricyclic antidepressant as monotherapy. Nine (16 %) were taking combination treatments. Six (7 %) patients were admitted acutely at the for in-patients treatment due to pain exacerbations. None were treated with intravenous fosphenytoin.

### Collection of scientific data

Scientific data were collected on all the included patients. This data collection has made it possible to describe demographics of the included patients in this paper as well as the clinical characteristics and neuroanatomical abnormalities of our patient population, which so-far has resulted in five publications [11, 12, 14–16].

## Discussion

The most important experiences from the implementation of the described cross-speciality TN management program were that it proved to be feasible to standardize the referral pathway and work-up, that the program ensured acceptable waiting times, and that early high-quality MRI ensured correct diagnosis and a standardized basis before decision-making on impending surgery. Moreover, the referral of the subgroup of patients in need for surgery was not unacceptably prolonged and the management program ensured continuous evaluation of pharmacological and surgical management, and provides the basis for scientific research. The described program has also facilitated centralization of expertise in TN and a close collaboration between the involved neurological, neurosurgical and neuroradiological departments ensures a higher volume of TN patients per clinician. The importance of this is supported by Kalkanis et al. who documented that the morbidity rate was lower at specialized high-volume surgical centres [17].

### Reduction of waiting time

Because TN is one of the most painful diseases known to mankind a short waiting time for out-patient work-up and treatment is important. Early diagnosis will save some TN patients from unnecessary dental treatments [16] and years of suboptimal medical treatment. Median waiting time for the first out-patient visit was 42 and 37 days for subsequent MRI, which we consider acceptable in a public health care system. It is our clinical experience that the waiting time for first out-patient visit has been reduced significantly since implementation. Likewise it is our clinical experience that the waiting time has been lower and the quality of the MRI description higher after implementation of the program. The specific TN protocol for the 3.0 Tesla MRI scans not only enables the neurologist to ensure correct diagnosis and rule out symptomatic causes of pain, but also gives the neurosurgeon a standardized basis for decision-making before impending neurosurgery. Based on feedback from TN patients and patient’s organizations the reduction of waiting time has led to higher patient satisfaction.

Prior to the implementation of the program we did not register waiting time or patient satisfaction specifically for TN patients, so we cannot provide scientific documentation for improvement of these parameters after implementation of the program. However, with the described structured and accelerated manner of referral we demonstrate a relatively short and acceptable waiting time for diagnosis and imaging.

### When are the medical intractable patients referred to neurosurgery?

The median time from the first out-patient visit at DHC to referral to neurosurgery was approximately 2 months for all patients but only 20 days for patients who were initially referred to neurosurgery and then re-directed to DHC for pre-surgical evaluation. This delay must be considered acceptable given that the patients on average had suffered from TN for 7 years and considering that even among those initially referred for surgery and then re-directed to DHC for pre-surgical evaluation, half could be sufficiently controlled be medical management. Thus, the accelerated work-up and treatment program ensures that patients with medically intractable pain are quickly referred to neurosurgery. In addition, for the patients who were initially referred to neurosurgery, who could be controlled on medical treatment, the re-direction to DHC reduced their waiting time for optimization of medical treatment and ensured that these patients did not undergo neurosurgery with its potential complications.

With the limited scientific evidence regarding efficacy of medical treatment [18–20], there is no single answer as to how many medications should be tried out before a TN patient is deemed medically refractory and surgery should be considered. Moreover, there is a lack of well-designed neurosurgical studies using independent evaluators of the efficacy and complications of microvascular decompression [21, 22], which makes the decision process even more challenging.

### Mono- or combination therapy

The international guidelines [13] on TN treatment recommend carbamazepine and oxcarbazepine as first line treatment based on clinical studies [5, 6, 23, 24]. Other drugs used to treat TN have not been investigated to the same extent but some smaller studies showed promising results using pregabalin [25], lamotrigine [26], baclofen [27] and gabapentin [28]. In the international guidelines it is stated that “if any of these sodium-channel blockers (carbamazepine or oxcarbazepine, edt.) are ineffective, referral for a surgical consultation would be a reasonable next step” [13]. However, the guidelines also state that “considering the relatively narrow mechanism of action of the available drugs (carbamazepine, edt.), combination treatments might be useful” [13].

Based on our clinical experience we agree with the international treatment guidelines although we find that referral for neurosurgery after failed monotherapy may be too hasty and we in general try out a combination treatment before referral to surgery. Unfortunately, the scientific support for combination treatment is sparse and there are no published studies directly comparing monotherapy with polytherapy [29]. We suggest that follow up on medical treatment should remain in the hands of experts until the condition is stable and the patient is familiar with the program of titrating up and tapering of medication according to the level of pain and side effects. We suggest that 2 years of follow up is appropriate, but this depends on the resources of the clinic and the health care system.

### Collection of scientific data and methodological considerations

The described structured management program has made it possible to prospectively collect scientific data, which so far has resulted in five publications, while several other manuscripts are in preparation. This is an important advantage of the systematic approach to patient management, since there is a huge need for scientific research in TN both with respect to controlled drug trials as well as to determine the optimal time for referral to surgery, i.e., which and how many drugs should be tried before surgery should be considered. To meet this need for evidence we are currently prospectively following a large representative population of TN patients at DHC to document efficacy, side effects, complications and patient satisfaction after medical and surgical treatment in an open label design. The outlined management program is not based on scientific evidence but on clinical experience which is a limitation. Although the presented data are not evidence based, we consider it important to describe our management program due to the lack of prior reports on how to structure TN management in clinical practice. We suggest this description of the management as a starting point from which to make adjustments, start discussions and collect scientific evidence on treatment efficacy and patient satisfaction.

## Conclusions

According to initial feedback from patients and clinicians, the newly implemented accelerated cross-speciality management program represents an improvement of our TN management by means of an acceptable waiting time, fast diagnosis and high-quality neuroimaging by specialists and standardized treatment and information to patients. We demonstrate that a formal collaboration across medical, diagnostic and surgical specialties is feasible at a regional and national level. Furthermore, we show that enrolment in a structured management program like this does not hold patients with medically intractable pain back for unacceptably long time before referral to neurosurgery and that the medical treatment is properly tested before impending neurosurgery. In our opinion, this is crucial to secure a high quality of TN management. We encourage other centres to publish their experiences with structured management programs in TN and to collect scientific evidence for the efficacy and side effects of medical and surgical treatment.
